# Data on changes in travel destination preferences of Thai domestic travelers before and after the COVID-19 pandemic

**DOI:** 10.1016/j.dib.2023.109202

**Published:** 2023-05-04

**Authors:** Sirin Prommakhot, Tosporn Arreeras, Mikiharu Arimura

**Affiliations:** a School of Management, Mae Fah Luang University, Chiang Rai 57100, Thailand; b Urban Safety Innovation Research Group, Mae Fah Luang University, Chiang Rai 57100, Thailand; c Division of Sustainable and Environmental Engineering, Muroran Institute of Technology, Muroran 050-8585, Japan

**Keywords:** Domestic tourism, Destination choices, Behavioral changes, Post-pandemic tourism, Travel behaviors

## Abstract

This data article analyzes the changes in travel habits and destination preferences among Thai domestic travelers before and after the COVID-19 pandemic. The data was collected through an online survey conducted on Facebook, Line, and Instagram, with a sample size of 460 valid respondents. The article provides descriptive statistics and frequency data on travel behavior and attitudes related to various tourist attractions before and after the onset of the pandemic. These insights can be valuable for transportation and tourist destination management in Thailand, as they can be used to compare with other studies using similar methods and outcomes and help to develop specialized and targeted solutions for addressing changes in travel trends and demand after the pandemic. For more information, see the full article titled “Using factor analyses to understand the post-pandemic travel behavior in domestic tourism through a questionnaire survey.”

**Specifications Table**

**Table:** 

Subject	Tourism
Specific subject area	Travel Behavior; Tourist Attractions
Type of data	Tables
How the data were acquired	Online survey through a researcher-designed electronic questionnaire.
Data format	Raw Descriptive Analyzed
Description of data collection	A Google Forms-based electronic survey was created and shared via email and social networking services (SNS) in September and October of 2021. The survey collected data on participants' demographic information, travel behaviors before and after the pandemic, and travel satisfaction. The target respondent for the survey was Thai tourists who had visited domestic tourist destinations. Out of 462 survey participants, 460 valid responses were included in the subsequent analysis after incomplete and unanswered surveys were removed.
Data source location	· City/Town/Region: All regions · Country: Thailand · Latitude and longitude: 15.870032, 100.992541
Data accessibility	Repository name: Mendeley Data [1] Data identification number: 10.17632/jj4mzgzv4k.3 Direct URL to data: https://doi.org/10.17632/jj4mzgzv4k.3
Related research article	Chansuk, C., Arreeras, T., Chiangboon, C., Phonmakham, K., Chotikool, N., Buddee, R., Pumjampa, S., Yanasoi, T., & Arreeras, S. (2022). Using factor analyses to understand the post-pandemic travel behavior in domestic tourism through a questionnaire survey. *Transportation Research Interdisciplinary Perspectives, 16*, 100,691. https://doi.org/10.1016/j.trip.2022.100691[2]

**Value of the Data**

• Provides valuable insights for businesses in the travel and tourism industry, including travel agencies, airlines, hotels, and restaurants, to adjust their offerings and marketing strategies based on changing travel preferences.
• Offers policymakers and government agencies a better understanding of how to support the recovery of the travel and tourism industry in Thailand, as well as potentially in other countries.
• Provides an opportunity for researchers to explore the factors that influence domestic travel behavior and preferences, which can inform future studies on consumer behavior and decision-making.
• Provides a unique and valuable dataset for researchers and students to analyze, interpret, and use in further research projects and studies, potentially contributing to new discoveries in the field of tourism and hospitality.

## 1 Objective

The COVID-19 pandemic has had a significant impact on the travel industry [3], and it is crucial to understand how it has influenced the destination preferences of domestic travelers in Thailand. This research aims to delve into the data on the changes in travel destination preferences of Thai domestic travelers before and after the COVID-19 pandemic. By conducting a thorough analysis of this data, we hope to gain insight into the factors that shape the decision-making process of Thai domestic travelers when it comes to choosing a travel destination during times of crisis [[4], [5]]. This research will not only provide valuable information for the development of targeted tourism marketing strategies, but it will also help to shed light on the impact of the pandemic on the travel industry as a whole. Ultimately, this research aims to contribute to a greater understanding of the evolving preferences and behavior of domestic travelers in Thailand before and after the COVID-19 pandemic.

## 2 Data Description

The survey aimed to address the changes in travel destination preferences of Thai tourists for domestic tourist destinations pre- and post- COVID-19 pandemic, as well as the travel decision-making process post-pandemic. The structure of survey questionnaire was based on prior research [2]. The 460 valid responses were distributed through email and social networking site (SNS) platforms. An example of questionnaire survey is available on the dataset in Mendeley Data repository [1].

The questionnaire survey instrument including:

• Socio-demographics information: gender, age group, level of education, occupation, monthly income, vehicle ownership, household size, marital status, and current residence.
• Travel destination before and after COVID-19 pandemic:
• Environment destination (e.g., coastal, forest, mountain)
• Historical destination (e.g., castles, temple, museum)
• Cultural destination (e.g., festivals and cultural events, floating market)
• Recreational destination (e.g., theme parks, sports and outdoors activities, entertainment destinations)
• Ethnic destination (e.g., homestay, workshop activities, Karen village)
• Business destination (e.g., conference, seminar, meeting, business negotiation)
• Travel decision-making for preference destination after COVID-19 pandemic: “I'd love to!”, “I'm not sure”, and “Definitely not!”.

The descriptive analysis of the dataset is presented in three tables, which provide insight into the frequency of travel destination preference of Thai tourists for domestic destinations before and after the COVID-19 pandemic. Table 1 presents the frequency of travel destination preference before the COVID-19 pandemic. Table 2 presents the frequency of travel destination preference after the COVID-19 pandemic. Table 3 presents the perspectives on travel decision-making of the respondents in the post-COVID-19 pandemic. Additionally, a list of the available files in the dataset are presented in Table 4. The full survey and its analysis are available in Mendeley Data repository.

**Table 1:** The proportion travelers before COVID-19 pandemic.

		Frequency of travel destination (%)					
Variable	Frequency (%)	Environment	Historical	Cultural	Recreational	Ethnic	Business
**Total**	460 (100.0)	73.7	7.8	3.7	14.3	0.2	0.2
**Gender**							
Male	100 (21.7)	83.0	7.0	4.0	5.0	1.0	0.0
Female	340 (73.9)	70.6	8.2	3.2	17.6	0.0	0.3
Self-identity	20 (4.3)	80.0	5.0	10.0	5.0	0.0	0.0
**Age group (years)**							
<20	56 (12.2)	64.3	3.6	7.1	25.0	0.0	0.0
21 - 30	245 (53.3)	74.3	5.7	2.4	17.1	0.4	0.0
31 - 40	31 (6.7)	71.0	16.1	3.2	9.7	0.0	0.0
41 - 50	80 (17.4)	80.0	11.3	1.3	7.5	0.0	0.0
51 - 60	40 (8.7)	80.0	7.5	7.5	2.5	0.0	2.5
Over 60	8 (1.7)	37.5	37.5	25.0	0.0	0.0	0.0
**Level of education**							
Under bachelor's	106 (23.0)	69.8	7.5	2.8	19.8	0.0	0.0
Bachelor's	320 (69.6)	74.4	7.8	3.8	13.4	0.3	0.3
Above bachelor's	34 (7.4)	79.4	8.8	5.9	5.9	0.0	0.0
**Occupation**							
Student	236 (51.3)	72.5	5.1	3.4	18.6	0.4	0.0
Business owner	35 (7.6)	71.4	8.6	2.9	14.3	0.0	2.9
Officer	40 (8.7)	77.5	10.0	5.0	7.5	0.0	0.0
Employee	68 (14.8)	82.4	7.4	2.9	7.4	0.0	0.0
Homemaker	6 (1.3)	83.3	0.0	0.0	16.7	0.0	0.0
Worker	25 (5.4)	72.0	8.0	4.0	16.0	0.0	0.0
Farmer	4 (0.9)	75.0	25.0	0.0	0.0	0.0	0.0
Others	46 (10.0)	65.2	19.6	6.5	8.7	0.0	0.0
**Monthly income (THB)**							
≤ 15,000	266 (57.8)	69.9	7.5	3.4	18.8	0.4	0.0
15,000 - 30,000	123 (26.7)	75.6	9.8	3.3	10.6	0.0	0.8
30,001 - 45,000	29 (6.3)	86.2	6.9	3.4	3.4	0.0	0.0
45,001 - 60,000	23 (5.0)	91.3	4.3	4.3	0.0	0.0	0.0
> 60,000	19 (4.1)	73.7	5.3	10.5	10.5	0.0	0.0
**Vehicle ownership**							
Yes	349 (75.9)	77.4	8.0	3.2	11.2	0.0	0.3
No	111 (24.1)	62.2	7.2	5.4	24.3	0.9	0.0
**Household size (including the respondent)**							
1	16 (3.5)	75.0	12.5	6.3	6.3	0.0	0.0
2 - 3	137 (29.8)	73.7	10.9	1.5	13.9	0.0	0.0
4 - 5	248 (53.9)	73.0	6.9	4.4	14.9	0.4	0.4
> 5	59 (12.8)	76.3	3.4	5.1	15.3	0.0	0.0
**Marital status**							
Unmarried	331 (72.0)	73.1	6.3	3.3	16.9	0.3	0.0
Married	94 (20.4)	76.6	11.7	6.4	4.3	0.0	1.1
Not mentioned	35 (7.6)	71.4	11.4	0.0	17.1	0.0	0.0
**Current residence (region)**							
Central	210 (45.7)	73.8	8.6	1.4	15.7	0.5	0.0
Northern	134 (29.1)	79.1	9.0	2.2	9.0	0.0	0.7
Southern	78 (17.0)	66.7	3.8	11.5	17.9	0.0	0.0
North-Eastern	12 (2.6)	66.7	16.7	0.0	16.7	0.0	0.0
Eastern	21 (4.6)	66.7	4.8	4.8	23.8	0.0	0.0
Western	5 (1.1)	80.0	0.0	20.0	0.0	0.0	0.0

**Table 2:** Travel destination after COVID-19 pandemic.

		Frequency of travel destination (%)					
Variable	Frequency (%)	Environment	Historical	Cultural	Recreational	Ethnic	Business
**Total**	460 (100.0)	81.1	2.8	3.5	10.2	2.0	0.4
**Gender**							
Male	100 (21.7)	86.0	3.0	4.0	6.0	1.0	0.0
Female	340 (73.9)	79.4	2.9	3.5	11.8	1.8	0.6
Self-identity	20 (4.3)	85.0	0.0	0.0	5.0	10.0	0.0
**Age group (years)**							
<21	56 (12.2)	66.1	0.0	5.4	25.0	3.6	0.0
21 - 30	245 (53.3)	82.0	1.2	2.4	12.7	1.2	0.4
31 - 40	31 (6.7)	80.6	3.2	9.7	6.5	0.0	0.0
41 - 50	80 (17.4)	92.5	3.8	2.5	0.0	1.3	0.0
51 - 60	40 (8.7)	80.0	10.0	2.5	0.0	5.0	2.5
Over 60	8 (1.7)	50.0	25.0	12.5	0.0	12.5	0.0
**Level of education**							
Under bachelor's	106 (23.0)	82.1	2.8	3.8	8.5	0.9	1.9
Bachelor's	320 (69.6)	80.3	2.8	2.8	11.6	2.5	0.0
Above bachelor's	34 (7.4)	85.3	2.9	8.8	2.9	0.0	0.0
**Occupation**							
Student	236 (51.3)	78.4	0.8	3.8	15.3	1.7	0.0
Business owner	35 (7.6)	91.4	5.7	0.0	2.9	0.0	0.0
Officer	40 (8.7)	87.5	5.0	5.0	2.5	0.0	0.0
Employee	68 (14.8)	85.3	1.5	1.5	7.4	2.9	1.5
Homemaker	6 (1.3)	100.0	0.0	0.0	0.0	0.0	0.0
Worker	25 (5.4)	84.0	8.0	0.0	4.0	0.0	4.0
Farmer	4 (0.9)	50.0	0.0	0.0	0.0	50.0	0.0
Others	46 (10.0)	73.9	8.7	8.7	6.5	2.2	0.0
**Monthly income (THB)**							
≤ 15,000	266 (57.8)	79.3	2.3	2.6	13.5	1.9	0.4
15,000 - 30,000	123 (26.7)	81.3	2.4	5.7	7.3	2.4	0.8
30,001 - 45,000	29 (6.3)	89.7	0.0	6.9	3.4	0.0	0.0
45,001 - 60,000	23 (5.0)	95.7	4.3	0.0	0.0	0.0	0.0
> 60,000	19 (4.1)	73.7	15.8	0.0	5.3	5.3	0.0
**Vehicle ownership**							
Yes	349 (75.9)	82.5	2.9	2.9	9.5	1.7	0.6
No	111 (24.1)	76.6	2.7	5.4	12.6	2.7	0.0
**Household size (including the respondent)**							
1	16 (3.5)	75.0	12.5	6.3	0.0	6.3	0.0
2 - 3	137 (29.8)	81.0	2.9	3.6	10.9	1.5	0.0
4 - 5	248 (53.9)	81.5	1.6	3.2	10.9	2.4	0.4
> 5	59 (12.8)	81.4	5.1	3.4	8.5	0.0	1.7
**Marital status**							
Unmarried	331 (72.0)	78.9	2.1	3.9	13.0	1.8	0.3
Married	94 (20.4)	88.3	5.3	2.1	0.0	3.2	1.1
Not mentioned	35 (7.6)	82.9	2.9	2.9	11.4	0.0	0.0
**Current residence (region)**							
Central	210 (45.7)	88.1	1.0	2.4	6.7	1.9	0.0
Northern	134 (29.1)	77.6	3.0	3.7	11.9	3.0	0.7
Southern	78 (17.0)	69.2	6.4	6.4	15.4	1.3	1.3
North-Eastern	12 (2.6)	83.3	8.3	0.0	8.3	0.0	0.0
Eastern	21 (4.6)	76.2	0.0	4.8	19.0	0.0	0.0
Western	5 (1.1)	80.0	20.0	0.0	0.0	0.0	0.0

**Table 3:** Do you still want to travel after the end of the COVID-19 pandemic?.

		Frequency of travel decisions (%)		
Variable	Frequency (%)	I'd love to!	I'm not sure	Definitely not!
**Total**	460 (100.0)	75.2	23.9	0.9
**Gender**				
Male	100 (21.7)	74.0	25.0	1.0
Female	340 (73.9)	76.2	23.2	0.6
Self-identity	20 (4.3)	65.0	30.0	5.0
**Age group (years)**				
< 21	56 (12.2)	71.4	26.8	1.8
21 - 30	245 (53.3)	76.7	22.4	0.8
31 - 40	31 (6.7)	71.0	29.0	0.0
41 - 50	80 (17.4)	75.0	25.0	0.0
51 - 60	40 (8.7)	75.0	22.5	2.5
Over 60	8 (1.7)	75.0	25.0	0.0
**Level of education**				
Under bachelor's	106 (23.0)	64.2	35.8	0.0
Bachelor's	320 (69.6)	77.5	21.6	0.9
Above bachelor's	34 (7.4)	88.2	8.8	2.9
**Occupation**				
Student	236 (51.3)	75.4	23.3	1.3
Business owner	35 (7.6)	77.1	22.9	0.0
Officer	40 (8.7)	80.0	20.0	0.0
Employee	68 (14.8)	80.9	17.6	1.5
Homemaker	6 (1.3)	66.7	33.3	0.0
Worker	25 (5.4)	56.0	44.0	0.0
Farmer	4 (0.9)	100.0	0.0	0.0
Others	46 (10.0)	69.6	30.4	0.0
**Monthly income (THB)**				
≤ 15,000	266 (57.8)	71.8	27.4	0.8
15,000 - 30,000	123 (26.7)	74.8	24.4	0.8
30,001 - 45,000	29 (6.3)	89.7	10.3	0.0
45,001 - 60,000	23 (5.0)	91.3	4.3	4.3
> 60,000	19 (4.1)	84.2	15.8	0.0
**Vehicle ownership**				
Yes	349 (75.9)	75.6	23.8	0.6
No	111 (24.1)	73.9	24.3	1.8
**Household size (including the respondent)**				
1	16 (3.5)	50.0	43.8	6.3
2 - 3	137 (29.8)	75.9	24.1	0.0
4 - 5	248 (53.9)	77.0	21.8	1.2
> 5	59 (12.8)	72.9	27.1	0.0
**Marital status**				
Unmarried	331 (72.0)	74.0	25.1	0.9
Married	94 (20.4)	78.7	21.3	0.0
Not mentioned	35 (7.6)	77.1	20.0	2.9
**Current residence (region)**				
Central	210 (45.7)	77.1	21.9	1.0
Northern	134 (29.1)	74.6	24.6	0.7
Southern	78 (17.0)	71.8	26.9	1.3
North-Eastern	12 (2.6)	66.7	33.3	0.0
Eastern	21 (4.6)	71.4	28.6	0.0
Western	5 (1.1)	100.0	0.0	0.0

**Table 4:** List of available files in the dataset.

File name	Description	Format
Travel_Dataset	The complete dataset analyzed a dataset comprising responses from 460 participants in a nationwide survey in this study. The dataset includes a variable header, which identifies the coded variables (X1 through X12) and the responses from each individual survey participant.	.csv
Dataset Description	A comprehensive description of the dataset used in this study is provided. The dataset comprises the survey questions as presented to the participants, along with the corresponding numerical coding of the responses within the dataset.	.pdf
Questionnaire Form	The questionnaire was divided into 4 sections, consists of socio-demographics information, travel destination before the COVID-19 pandemic, travel destination after the COVID-19 pandemic, and travel decision-making for preference destination after the pandemic.	.pdf

## 3 Experimental Design, Materials and Methods

This data is providing an additional descriptive analysis of the dataset based on a related article [2]. The dataset employed an online survey methodology to gather data from Thai tourists who had visited domestic tourist destinations. The survey was administered via Google Forms and disseminated through various digital channels, including email and social media platforms such as Facebook, Line, and Instagram. The survey was conducted during the months of September and October 2021. In order to accurately estimate an appropriate sample size for the dataset, we employed the Taro Yamane formula, which offers a straightforward approach to determining sample size based on maximum variability and a 95 percentile of confidence level [6]. The minimum number of surveys required for the study was calculated to be 400. Ultimately, we collected 462 responses and, after eliminating incomplete or unanswered questionnaires, a total of 460 valid responses were included in the final analysis. The collected data were analyzed using Statistical Package for Social Science (SPSS) version 28.0. Descriptive statistics were utilized to examine the demographics of the participants.

The limitation from this data has been identified. The majority of respondents are students, which could suggest that the sample may not accurately reflect the characteristics of the broader population. This is because the survey was distributed through Social Networking Site (SNS), which students are more likely to be active on social media platforms, leading to a higher likelihood of their participation in the survey. Additionally, the survey through SNS is implied as random convenience sampling, which can lead to biased results as individuals who self-select themselves to participation. It may have different characteristics than those who do not participate in the survey. As the regulation of social distancing was still active in Thailand during data collection, we utilized social networking sites (SNS) as an effective solution for collecting questionnaire survey responses. However, a longer period of time and a larger budget for data collection would likely result in greater distribution and help balance any potential biases. Moreover, the sample population is recommended to be proportional distribution to avoid any biases. However, despite these limitations, this dataset still provides valuable insights into the preferences of Thai tourists for domestic travel destinations before and after the COVID-19 pandemic. Acknowledging these limitations is crucial to understand the potential impact on the findings and consider its implications for future studies.

## Ethics Statements

Our study adheres to rigorous ethical standards, having fully addressed all ethical considerations within the research design. The methodology employed is a survey alone, and thus, the requirement for prior ethics approval is obviated. Participants were fully apprised of their rights and provided their informed consent. To ensure anonymity and protect the privacy of individual respondents, any identifying information has been purged from our dataset.

## CRediT authorship contribution statement

**Sirin Prommakhot:** Writing – original draft, Data curation. **Tosporn Arreeras:** Conceptualization, Methodology, Supervision, Writing – review & editing, Validation. **Mikiharu Arimura:** Investigation, Supervision.

## Declaration of Competing Interest

The authors declare that they have no financial interests or personal relationships that could potentially influence the research presented in this paper.

## Data Availability

Dataset on changes in travel destination preferences of Thai domestic travelers during the COVID-19 pandemic (Original data) (Mendeley Data).
